# Linking growth pattern with Lipprints and fingerprints

**DOI:** 10.6026/973206300222348

**Published:** 2026-04-30

**Authors:** Akshay E.R, Manjunath Hegde, Chaitra C, Vinaya S Pai, Mrunmayee Gholap

**Affiliations:** 1 Department of Orthodontics and Dentofacial Orthopedics, Bangalore Institute of Dental Sciences, Bangalore, Karnataka, India

**Keywords:** Cheiloscopic, lip print, dermatoglyphic, fingerprint, growth pattern, Jarabak, Galton, Suzuki

## Abstract

Lips have a significant role in aesthetic as well as identification purpose and needs to be taken into account for human identification. Therefore, it is of interest to establish a correlation between cheiloscopic patterns (Lip print), dermatoglyphic patterns (Fingerprint) and different growth patterns. The cheiloscopic pattern was obtained using a dark colored lip stick and transparent adhesive tape. Suzuki and Tsuchihashi's classification was used to classify the cheiloscopic patterns. Finger prints were recorded using inkless stamp pads onto white sheets and. There was a statistical significance only between loop pattern of fingerprints in females who had horizontal growth pattern (p= 0.04). Thus, we show that the dermatoglyphic pattern, cheiloscopic pattern and growth pattern are not correlated with each other.

## Background:

The most important esthetic goal of orthodontics is to achieve a balanced smile of which lips play a crucial role and at the same time it acts as important tool for human identification in civil and criminal issues [1]. The biological phenomenon of system of furrows on the red part of the human lips was first noted and described by RS Fischer, an anthropologist in 1902 [2]. Cheiloscopy deals with examination of systems of furrows on the red part of human lips. It was in Hungary in 1961 that the first research in Europe was carried out on the subject of lip prints [3]. Suzuki and Tsuchihashi established that the arrangement of lines on the lips is individual and unique for each human being and remains unchanged during life just like fingerprints [4]. Friction ridge skin on the fingers and palms are known to be physical characteristic of an individual that does not change over time. The study of pattern of fingerprints is known as dermatoglyphics. The epidermal ridges on the lateral fingertips are usually laid down between 10 and 18 weeks of gestation and remain unchanged except for an increase in size in parallel with general growth [5, 6]. Study by Nanda and Ghosh have clearly shown that the dynamic changes in dental, skeletal and facial integument occur over the entire period of active growth and even into decades past the age of 20 years [7]. The basis of studying the relationship between dermatoglyphics and various dental anomalies is due to development of teeth and associated structures which coincides with development of epidermal ridges during 6 to 13 weeks of intrauterine life. This also coincides with the development of lips. A Study by Adamu *et al.* assessed the correlation between lip print pattern and skeletal malocclusion in anteroposterior plane [8]. Therefore, it is of interest to establish a correlation between cheiloscopic patterns, dermatoglyphic pattern and different growth patterns since no such study was found in the literature for the same.

## Materials and Methods:

The sample group consisted of pretreatment lateral cephalograms of 90 untreated individuals and cheiloscopic patterns and dermatoglyphic patterns of the same individuals. The lateral cephalograms were obtained from the Department of Orthodontics and Dentofacial Orthopedics, Bangalore Institute of Dental Sciences and Hospital and Post Graduate Research Centre. In this study group, out of ninety samples, 30 had horizontal growth pattern, 30 had vertical growth pattern and 30 had average growth pattern based on Jarabak ratio.

## Inclusion criteria:

Patients with

[1] No previous history of orthodontic treatment.

[2] No history of trauma to the face or fingers

[3] Full complement of permanent teeth irrespective of third molar.

[4] Good quality of lateral cephalograms

## Exclusion criteria:

[1] Congenital cranio-facial deformities.

[2] A history of forceps assisted birth.

[3] Bone deformities.

[4] Patients with injuries to the pads of finger or lip

The nature of the study was explained to the patient and informed consent was taken following which the dermatoglyphic pattern and cheiloscopic patterns were recorded. Descriptive analysis of all the explanatory variables was done using frequency and proportions for each group. Inferential statistics i.e., Chi Square Test was used to compare the Fingerprint and Lip print patterns based on Growth patterns and based on gender in each growth pattern group. The level of significance was set at p<0.05.

## Results:

In the frequency distribution of fingerprint patterns observed in subjects with different growth patterns (Table 1 - see PDF), distinct trends emerged across the horizontal, average and vertical growth patterns. Among the L5 finger, the loop pattern predominated across all growth patterns, accounting for 80.0% (n=24) in both horizontal and average categories and slightly lower at 73.3% (n=22) in the vertical group. In the L4 finger, the whorl pattern dominated, particularly in the average growth pattern, where it constituted 66.7% (n=20). Regarding the L3 finger, the loop pattern was observed most frequently across all classifications, highest in the average group at 60.0% (n=18), followed by 53.3% (n=16) in horizontal and 46.7% (n=14) in vertical classifications. For the L2 finger, the loop pattern remained prominent, accounting for 50.0% (n=15) in the average category, but slightly lower in horizontal and vertical classifications at 43.3% (n=13) and 30.0% (n=9), respectively. The whorl pattern showed increasing prevalence, highest in the vertical group at 50.0% (n=15), followed by 36.7% (n=11) in the average category and 26.7% (n=8) in the horizontal group. The arch pattern, similar to other fingers, was the least observed, highest in horizontal growth at 30.0% (n=9), followed by 20.0% (n=6) in vertical and lowest at 13.3% (n=4) in the average group. In the frequency distribution of fingerprint patterns for the right hand in subjects with different growth patterns, distinct variations were observed across horizontal, average and vertical classification (Table 2 - see PDF). For the R1 finger, the loop pattern showed considerable variation, with the highest frequency in the average group at 60.0% (n=18), followed by 46.7% (n=14) in the vertical group and 40.0% (n=12) in the horizontal group. Regarding the R2 finger, the loop pattern was consistently observed at 50.0% (n=15) across both horizontal and average groups but decreased to 36.7 %( n=11) in the vertical group. For the R3 finger, the loop pattern remained dominant across all classifications, highest at 73.3% (n=22) in horizontal, followed by 70.0% (n=21) in average and slightly lower at 63.3% (n=19) in the vertical group. In the R4 finger, the whorl pattern was dominant, with the highest frequency observed in vertical group at 66.7% (n=20), followed by 63.3% (n=19) in the average classification and 43.3% (n=13) in horizontal. Lastly, the R5 finger exhibited a strong presence of the loop pattern, observed most frequently in horizontal subjects at 76.7 %( n=23), followed by 63.3% (n=19) in average and 60.0% (n=18) invertical group. In the comparison of predominant fingerprint patterns across different growth patterns, notable trends were observed in both hands (Table 3 - see PDF). For the left hand, the loop pattern remained the most prevalent across all growth patterns, with identical proportions of 56.7% (n=17) in both horizontal and average groups, while a slightly lower presence was observed in the vertical group at 43.3% (n=13). Regarding the right hand, the loop pattern was dominant, highest in the average group at 63.3% (n=19), followed by 56.7% (n=17) in the horizontal group and 46.7% (n=14) in vertical group. When considering the overall distribution, the loop pattern maintained the highest frequency across all groups, observed at 66.7% (n=20) in horizontal group, decreasing to 56.7% (n=17) in the average group and further to 46.7% (n=14) in vertical group. In the comparison of lip print patterns among subjects with different growth patterns, distinct distributions were observed across horizontal, average and vertical groups (Table 4 - see PDF). Vertical grooves were found most frequently in subjects with an average growth pattern, accounting for 33.3% (n=10). The branched grooves exhibited an increasing trend, occurring least frequently in horizontal subjects at 16.7% (n=5), while their prevalence rose to 23.3% (n=7) in the average group and was highest at 33.3% (n=10) in vertical group. Intersected grooves showed variable distribution, with the highest frequency in horizontal subjects at 20.0% (n=6), followed by 13.3% (n=4) in vertical group and the lowest prevalence in the average group at 10.0 % (n=3). Reticular grooves maintained a consistent presence across all growth classifications, observed at an equal frequency of 30.09) in horizontal, average and vertical groups. The undifferentiated pattern was the least common across all growth patterns, appearing at 6.7% (n=2) in subjects of horizontal growth pattern and further decreasing to 3.3% (n=1) in both average and vertical groups. The statistical analysis indicated no significant association between lip print patterns and growth patterns, as demonstrated by a p-value of 0.83.

Growth patterns can be assessed using cephalometric analyses, particularly the mandibular plane angle, Y-axis, facial axis and SN-GoGn . angle in Jarabak ratio. These measurements help determine the vertical or horizontal tendency of facial growth [9]. Biometric identification has gained prominence in forensic and clinical sciences. Among various identifiers, fingerprints and lip prints have proven to be unique, stable and non-invasive. While fingerprints are well-established in forensic investigations, lip prints are emerging as potential identifiers in both forensic and dental fields. Both biometric traits develop during early intrauterine life and remain unchanged throughout a person's lifetime [10]. The cross-sectional study by Abdul *et al.* [11] investigated the potential correlation between fingerprint patterns and mandibular growth directions in individuals with a skeletal Class I relationship. The research involved 90 participants aged 20-35 years, categorized into three groups based on mandibular divergence: horizontal, average and vertical growers. Fingerprint patterns namely whorls, loops, arches were recorded and analysed for each subject. The pattern of all ten digits was analysed. The findings revealed a significant association between dermatoglyphic patterns and mandibular growth types. Specifically, individuals with horizontal growth patterns exhibited a higher prevalence of whorl patterns (80% in the left hand and 67% in the right hand), distinguishing them from average and vertical growers. In the current study, individuals were not segregated based on the sagittal skeletal discrepancy. This study found that in the horizontal group, loop pattern was more common females which are accordance with the above-mentioned study. Ball *et al.* [4] established that the arrangement of lines on the lips is individual and unique for each human being and remains unchanged during life, just like fingerprints. A study by Adamu *et al.* [8] presented statistically significant findings that reinforce the potential of lip print patterns as early diagnostic markers for skeletal malocclusion. In the study, lip prints were analyzed, particularly focusing on the middle third of the lower lip, where pattern clarity was highest. The results revealed a significant correlation (p < 0.05) between specific lip print types and malocclusion classes: Type I (clear vertical grooves) and Type II (branched grooves) were predominantly associated with Class I malocclusion, while Type III (intersected grooves) and Type IV (reticular patterns) were more frequently found in Class II malocclusion cases. These findings indicated a strong statistical association, suggesting that cheiloscopy could serve as a reliable, non-invasive screening tool in early orthodontic diagnosis. A study by Thete *et al.* [12] and Regan *et al.* [13] also showed that cheiloscopic patterns could be reliably used to predict skeletal malocclusion. There is no study in literature which correlated lip prints and growth pattern to make a reliable comparison. The results from the current study revealed distinct distributions of fingerprint and lip print patterns among subjects categorized based on different growth patterns. The frequency analysis provided insights into variations observed between horizontal, average and vertical growth classifications, along with gender-wise comparisons. The present study was done in an ethnically diverse metropolitan city. Future studies where the inclusion criteria are made more stringent by excluding subjects with the above mentioned local etiological factors and ethnic background might yield statistically significant results.

## Conclusion:

Data shows distinct distributions of fingerprint and lip print patterns across different growth classifications, with loop fingerprints predominating in horizontal and average growth patterns and a relative increase in whorl patterns among vertical growth patterns. Lip print analysis showed varied distributions, with vertical grooves more commonly seen in individuals with average growth patterns. Although these variations suggest possible associations of anthropological interest, no statistically significant correlation was found between fingerprint or lip print patterns and horizontal, vertical, or average growth patterns.
